# Super, Red Palm and Palm Oleins Improve the Blood Pressure, Heart Size, Aortic Media Thickness and Lipid Profile in Spontaneously Hypertensive Rats

**DOI:** 10.1371/journal.pone.0055908

**Published:** 2013-02-11

**Authors:** Chee-Meng Boon, Mei-Han Ng, Yuen-May Choo, Shiueh-Lian Mok

**Affiliations:** 1 Department of Pharmacology, Faculty of Medicine, University of Malaya, Kuala Lumpur, Malaysia; 2 Malaysian Palm Oil Board, Kajang, Selangor, Malaysia; University of Buenos Aires, Faculty of Medicine. Cardiovascular Pathophysiology Institute., Argentina

## Abstract

**Background:**

Oleic acid has been shown to lower high blood pressure and provide cardiovascular protection. Curiosity arises as to whether super olein (SO), red palm olein (RPO) and palm olein (PO), which have high oleic acid content, are able to prevent the development of hypertension.

**Methodology/Principal Findings:**

Four-week-old male spontaneously hypertensive rats (SHR) and Wistar-Kyoto (WKY) rats were fed 15% SO, RPO or PO supplemented diet for 15 weeks. After 15 weeks of treatment, the systolic blood pressure (SBP) of SHR treated with SO, RPO and PO were 158.4±5.0 mmHg (*p<0.001*), 178.9±2.7 mmHg (*p<0.001*) and 167.7±2.1 mmHg (*p<0.001*), respectively, compared with SHR controls (220.9±1.5 mmHg). Bradycardia was observed with SO and PO. In contrast, the SBP and heart rate of treated WKY rats were not different from those of WKY controls. The SO and PO significantly reduced the increased heart size and thoracic aortic media thickness observed in untreated SHR but RPO reduced only the latter. No such differences, however, were observed between the treated and untreated WKY rats. Oil Red O enface staining of thoracic-abdominal aorta did not show any lipid deposition in all treated rats. The SO and RPO significantly raised serum alkaline phosphatase levels in the SHR while body weight and renal biochemical indices were unaltered in both strains. Serum lipid profiles of treated SHR and WKY rats were unchanged, with the exception of a significant reduction in LDL-C level and total cholesterol/HDL ratio (atherogenic index) in SO and RPO treated SHR compared with untreated SHR.

**Conclusion:**

The SO, RPO and PO attenuate the rise in blood pressure in SHR, accompanied by bradycardia and heart size reduction with SO and PO, and aortic media thickness reduction with SO, RPO and PO. The SO and RPO are antiatherogenic in nature by improving blood lipid profiles in SHR.

## Introduction

Palm oil is rich in antioxidants and contains a high proportion of palmitic acid as well as considerable amount of oleic and linoleic acids [1] with oleic acid being the major unsaturated fatty acid that constituted up to 40% of the total fatty acid found in palm oil [2]. Technological advances in palm oil industry in the last two decades have resulted in the productions of super olein (SO) and red palm olein (RPO), which together with palm olein (PO) constitute the three major types of edible palm oils currently available commercially.

SO and PO are obtained from fractionation, refining, bleaching and deodorization of crude palm oil [1]. One major difference between these two oils is that SO, also known as double fractionated palm oil, is obtained by further fractionation of PO that resulted in an oil which is less saturated compared with PO. RPO has similar fatty acid composition as PO and is obtained by modifying the processing techniques of the crude palm oil which involve pretreatment followed by molecular distillation to produce deacidified and deodorized oil that retains as much as 80% of the original carotenoids in addition to a large amount of vitamin E [3], [4]. The fractionation process is the major turning point in modern palm oil industry as it brought about an increase in monounsaturated oleic acid with the simultaneous reduction in palmitic acid, the major saturated fatty acid found in palm oil [5].

Such increment in unsaturated fatty acid content in palm oil is desirable as epidemiological studies have demonstrated that dietary consumption of mono- and polyunsaturated fatty acids can decrease blood pressure (BP) and prevent the development of hypertension [6], [7]. Dietary supplementation with these fatty acids has provided evidence for their cardiovascular protective effects and their ability to lower the BP [6]. Oleic acid, an omega-9 monounsaturated fatty acid, recently receives substantial attention for its potential role in regulating the BP [8] since high oleic acid content in olive oil has been found to be responsible for the reduction in BP [9].

It has been suggested that the mechanism for the hypotensive effect of oleic acid is attributable to the increased concentration of oleic acid in lipid membranes that regulates the localization, activity and expression of important signaling molecules in the adrenergic receptor pathway, and enhances the production of vasodilatory stimuli while restricting the vasoconstriction pathways [9]. These findings may provide some evidence linking the high consumption of olive oil in the Mediterranean diet to its beneficial effect in maintaining a healthy BP level [10], [11].

We, therefore, postulated that SO, RPO and PO which have high oleic acid content may possess potential antihypertensive activity and cardiovascular protective action. Although SO, RPO and PO possess the same major fatty acids, there are slight differences in their fatty acid composition, principally in the unsaturated to saturated ratio due to their different processing methods. In this study, we wished to examine this hypothesis by evaluating the effects of dietary intake of SO, RPO and PO on the systolic blood pressure (SBP) and heart rate (HR) in the spontaneously hypertensive rats (SHR) and its normotensive Wistar-Kyoto (WKY) control strain of four weeks of age for a duration of 15 weeks. Heart weight and aortic media thickness were examined as they are the cardiovascular parameters that are directly associated with increased BP. Blood lipid profiles and biochemical indices of liver and renal function were performed to assess whether these parameters were affected after long-term dietary intake of these oils.

## Materials and Methods

### Ethics Statement and Animals

All experimental protocols involving animals were in agreement with and approved by the Animal Care and Use Committee, Laboratory Animal Science Centre, Faculty of Medicine, University of Malaya. Three-week-old male SHR and WKY rats were obtained from breeding pairs purchased from BioLASCO Taiwan Co. Ltd. Animals were housed individually in individually ventilated cage (IVC) fitted with individual HEPA filter and bed-o’cobs® Laboratory Animal Bedding under constant temperature of 23.0±1.0°C and subjected to a 12-hour dark/light cycle (lights on at 0700 h). All rats had *ad libitum* access to food and drinking water throughout the experiments.

### Animal Experiments

The SHR and WKY rats were both randomly divided into four groups (n = 6 per group) and each group was fed either a standard rat chow diet (controls), 15% SO supplemented diet, 15% RPO supplemented diet or 15% PO supplemented diet *ad libitum* for 15 weeks from 4 to 19 weeks of age. After 15 weeks of experiment, the animals were humanely sacrificed after a 12-hour fast by inhalation of an anesthetic agent, diethyl ether, achieved by placing the animal in a sealed container saturated with the anesthetic vapor. Blood samples were collected by exsanguination from anesthetized rats and centrifuged at 4000 rpm for 10 min. Serum was separated and stored in aliquots at −80°C until further analysis.

### Source and Formation of Diets

SO, RPO and PO were supplied by the Malaysian Palm Oil Board (MPOB) together with the analysis of their fatty acid and phytonutrient composition (Table 1). The fatty acids were determined as weight % as methyl esters. The 15% oil supplemented diets were formulated by mixing 15% wt/wt oil with standard rat diet (Specialty Feeds, Australia). The standard rat diet provides 4.240 kcal/kg (10.2% calories from fat, 18.9% calories from protein, 70.9% calories from carbohydrate) while the 15% supplemented diets provides 4.954 kcal/kg (34.7% calories from fat, 13.7% calories from protein, 51.6% calories from carbohydrate). The standard and formulated diets contained 0.18% sodium wt/wt and 0.15% sodium wt/wt, respectively.

**Table 1:** Fatty acid composition and phytonutrients of SO, RPO and PO.

	SO			RPO		PO	
**Fatty Acid (FA) (weight % as methyl esters)**							
Lauric Acid (C 12∶0, saturated)	0.3 (0.3)			0.0 (0.0)		0.2 (0.2)	
Myristic Acid (C 14∶0, saturated)	1.0 (1.0)			1.2 (1.2)		1.0 (1.0)	
Palmitic Acid (C 16∶0, saturated)	35.6 (35.0)			39.5 (39.5)		39.1 (40.3)	
Stearic Acid (C 18∶0, saturated)	3.9 (3.8)			3.7 (3.7)		3.9 (4.0)	
Eicosanoic/Arachidic Acid (C 20∶0, saturated)	0.3 (0.3)			0.0 (0.0)		0.4 (0.4)	
Palmitoleic Acid (C 16∶1, monounsaturated omega-7)	0.3 (0.3)			0.0 (0.0)		0.2 (0.2)	
Oleic Acid (C 18∶1, monounsaturated omega-9)	46.1 (45.4)			43.5 (43.4)		41.0 (42.2)	
Linoleic Acid (C18∶2, polyunsaturated omega-6)	13.8 (13.6)			12.2 (12.2)		10.9 (11.3)	
α-Linolenic Acid (C 18∶3, polyunsaturated omega-3)	0.3 (0.3)			0.0 (0.0)		0.4 (0.4)	
Saturated FA (%)	40.4		44.4		45.9		
Unsaturated FA (%)	59.6		55.6		54.1		
**Phytonutrients**							
Total Carotenes (mg/kg)	7.0			564.0		12.0	
Tocopherols and Tocotrienols (mg/kg)	188.0			1141.0		643.0	
Coenzyme Q10 (mg/kg)	0.0			6.0		0.0	

The amount of fatty acid expressed as a percentage is given in parenthesis.

SO = super olein; RPO = red palm olein; PO = palm olein.

### Measurement of Blood Pressure, Heart Rate, Food Intake and Body Weight

SBP was measured by non-invasive tail cuff method using the NIBP controller (AD Instruments, Australia) after the animals were pre-warmed at 38–40°C for 10 min [12], [13]. HR was monitored by the Cycle Variables Rate meter function which accurately detects and measures the tail pulse rate, and also directly indicates the position of the SBP point. Both SBP and HR as well as body weight were recorded before treatment (basal, week 0) and once weekly throughout the entire treatment period. Food intake was recorded daily and measured as calories consumed per day according to calculated nutritional parameter provided by the Specialty Feed, Australia.

### Determination of Blood Lipids and Biochemical Indices of Liver and Renal Function

Total serum cholesterol level was assayed using the cholesterol oxidase/peroxidase method, triglycerides was assayed using the lipase/glycerol kinase/glycerol phosphate oxidase-p-aminophenazone method, high density lipoprotein cholesterol (HDL-C) was determined by elimination method and low density lipoprotein cholesterol (LDL-C) was calculated using the formula, cholesterol - [(triglycerides/2.18)+HDL]. The serum was used to determine the biochemical indices of liver and renal function.

### Tissue Staining with Oil Red O and Hematoxylin-Eosin

The enface staining of the aorta with Oil Red O was used for gross determination of lipid deposition. The thoracic-abdominal aorta was cut open longitudinally with luminal surface facing up, pinned carefully on the surface of wax (Paraplast®) with stainless steel pins without stretching the tissue and fixed with 10% neutral buffered formalin. The inner aortic surface was stained with 0.3% Oil Red O (Sigma-Aldrich, USA) solution for 20 min at room temperature, and rinsed with 60% isopropanol and distilled water [14], [15].

A 2.5 mm transverse section of thoracic aorta below the aortic arch was harvested using a cutting instrument for general histopathological studies, routinely processed and embedded in paraffin. Paraffin sections (3 µm) were cut and fixed on glass slides. The tissue sections were stained with hematoxylin (SelecTech™, Surgipath®, USA) for 10 min, washed in running tap water, counterstained with eosin (SelecTech™, Surgipath®, USA) for 6 min and washed with running tap water. Stained sections were dehydrated in 95% ethanol (2×10 sec) and 100% ethanol (1×30 sec, 1×1 min). Slides were cleared in xylene (3×2 min) and were mounted with DPX mountant (Surgipath®, USA) and topped with coverslips. Prepared tissue sections were examined under a light microscope (Leica DM750).

### Data Analysis

Data are expressed as mean±SEM. The statistical analysis was performed using GraphPad Prism® 5 (GraphPad software). One-way or two-way ANOVA followed by Bonferroni’s multiple comparison test (where indicated) was used for statistical comparisons. Differences were considered statistically significant at *p<0.05*.

## Results

### Effect of Palm Oil Treatments on Systolic Blood Pressure and Heart Rate

**Figure 1:**
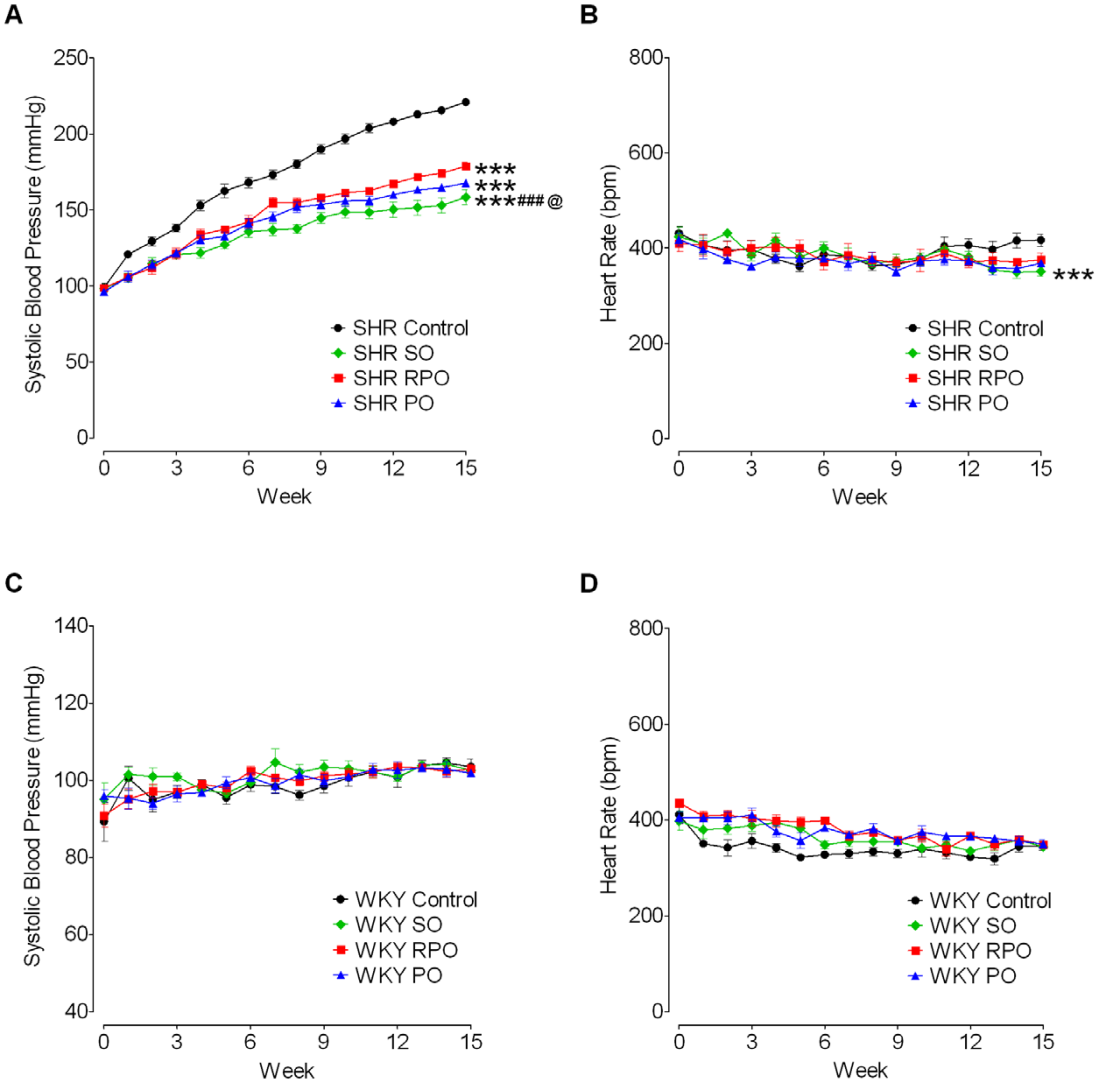
Time course of effect of oil supplementation on systolic blood pressure and heart rate. The graphs show the effect of super olein (SO), red palm olein (RPO) and palm olein (PO) supplemented diets on the systolic blood pressure (SBP) and heart rate (HR) in SHR and WKY rats from 4–19 weeks of age, at 0–15 weeks treatment period. SBP and HR of treated SHR and its controls (A and B, respectively). SBP and HR of treated WKY rats and its controls (C and D, respectively). Each point represents mean±SEM (n = 6). Two-way ANOVA was used for statistical comparisons between the different treated groups and their strain-matched controls on the SBP and HR changes over the whole duration of treatment. ***p<0.001, compared with SHR controls; ^###^ *p<0.001*, compared with RPO; ^@^ *p<0.05*, compared with PO.

The SO, RPO and PO markedly attenuated the rise in BP in the SHR over the whole duration of treatment (Fig. 1A). The SBP of SHR was significantly lower following treatments with SO (F_1,15_ = 42.38, *p<0.001*), RPO (F_1,15_ = 18.22, *p<0.001*) and PO (F_1,15_ = 29.80, *p<0.001*) compared with SHR controls, when the whole duration of treatment was assessed by two-way ANOVA. Meanwhile, the HR of SO treated SHR (F_1,15_ = 3.119, *p<0.001*), but not the PO (F_1,15_ = 0.0722) or RPO treated SHR (F_1,15_ = 0.1054), was significantly reduced compared with SHR controls (two-way ANOVA; Fig. 1B).

Before treatment, the SBP and HR of four-week-old SHR and WKY rats in the SO, RPO and PO groups were not significantly different from their strain-matched controls (Table 2). After 15 weeks of treatment (from 4–19 weeks of age), the SO, RPO and PO treated SHR had significantly lower SBP (*p<0.001*, in all cases) compared with SHR controls (Table 2). Within the palm oil treated groups, the SBP of SO treated SHR was significantly lower (*p<0.01*) than that of RPO treated SHR, but was not significantly different from that of PO treated SHR after 15 weeks of treatment. The SBP between PO and RPO treated SHR were also not significantly different from each other (Table 2). Similarly, the HR of SO, RPO and PO treated SHR were lower than that of SHR controls after 15 weeks of treatment, but significant differences were observed only for the SO (*p<0.01*) and PO treated groups (*p<0.05*) (Table 2).

**Table 2:** Systolic blood pressure and heart rate of rats before and after 15 weeks of treatment.

	SBP (mmHg)		HR (bpm)	
	Before	After	Before	After
**SHR**				
Control	98.9±3.0	220.9±1.5	430.9±15.2	416.8±12.5
SO	98.5±0.6^NS^	158.4±5.0***^,##^	424.1±20.7^NS^	350.8±10.1**
RPO	98.8±1.3^NS^	178.9±2.7***	409.8±16.8^NS^	375.7±13.7^NS^
PO	96.3±1.3^NS^	167.7±2.1***	418.2±12.9^NS^	368.7±3.1*
**WKY**				
Control	89.2±4.3	103.5±2.0	411.5±18.6	346.3±9.4
SO	95.1±4.3^NS^	102.6±1.3^NS^	398.1±19.8^NS^	344.3±7.7^NS^
RPO	90.8±3.0^NS^	103.0±1.1^NS^	435.5±7.0^NS^	349.0±6.3^NS^
PO	95.9±1.5^NS^	101.9±0.8^NS^	405.2±11.8^NS^	350.1±8.7^NS^

Values represent mean±SEM (n = 6).

NS , not significant;

* *p<0.05*,

** *p<0.01* and ***p<0.001, compared with strain-matched controls, and ^##^ *p<0.01*, compared with RPO, as assessed by Bonferroni's Multiple Comparison Test.SBP = systolic blood pressure; HR = heart rate; SO = super olein; RPO = red palm olein; PO = palm olein.

In contrast, the SO, RPO and PO did not produce any significant changes in the SBP and HR of WKY rats over the whole duration of treatment compared with WKY controls (Figs. 1C and 1D, respectively; Table 2).

### Food Intake and Body Weight

**Figure 2:**
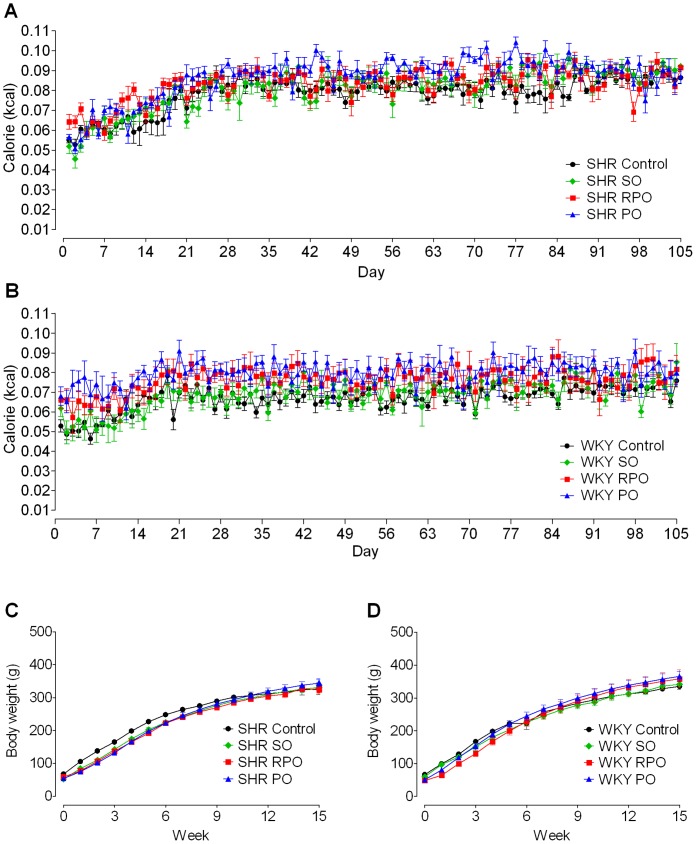
Calorie intake and body weight of treated and untreated SHR and WKY rats. The graphs show daily calorie intake and weekly body weight gain in SHR and WKY rats fed super olein (SO), red palm olein (RPO) and palm olein (PO) supplemented diets from 4–19 weeks of age, at 0–15 weeks of treatment period. Calorie intake of treated SHR and its controls (A), and treated WKY and its controls (B). Body weight of treated SHR and its controls (C), and treated WKY and its controls (D). Each point represents mean±SEM (n = 6).

All the treated and untreated SHR and WKY rats did not show any significant differences in their daily caloric intake expressed in kilocalorie (kcal) (Figs. 2A and 2B).

The average daily food intake for SHR controls was 18.49±0.56 g and SHR treated with SO, RPO and PO were 16.36±0.45 g, 16.78±0.43 g and 17.46±0.29 g, respectively. The average daily food intake for WKY controls was 15.76±0.57 g and WKY treated with SO, RPO and PO were 13.91±0.65 g, 15.40±0.96 g and 16.05±0.82 g, respectively.

The average daily sodium intake for SHR controls was 0.0333±0.0010 g and SHR treated with SO, RPO and PO were 0.0245±0.0007 g, 0.0252±0.0007 g and 0.0262±0.0004 g, respectively. The average daily sodium intake for WKY controls was 0.0284±0.0025 g and WKY treated with SO, RPO and PO were 0.0209±0.0009 g, 0.0231±0.0014 g and 0.0241±0.0012 g, respectively.

The body weights gained in treated animals over the 15 weeks treatment period were not significantly different from that of untreated animals (Figs. 2C and 2D). At the time of sacrifice, there were no significant differences in the mean body weight between the treated and untreated animals as well as within the treated groups (Table 3).

**Table 3:** Body weight, heart weight and aorta media thickness of rats after 15 weeks of treatment.

	Control	SO		RPO		PO	p_treatment_
**Body Weight (g)**							
SHR	327.7±18.4	337.1±10.0^NS^		328.7±13.5^NS^		346.3±11.7^NS^	NS
WKY	335.3±9.0	342.1±7.8^NS^		358.3±27.0^NS^		366.1±14.9^NS^	NS
**Heart (g)**							
SHR	1.60±0.11	1.45±0.04^NS^		1.45±0.04^NS^		1.38±0.04^NS^	NS
WKY	1.12±0.02	1.02±0.02^NS^		1.09±0.07^NS^		1.12±0.03^NS^	NS
**Heart/body (mg/g)**							
SHR	5.00±0.35	4.05±0.12*		4.44±0.12^NS^		4.00±0.07*	*<0.01*
WKY	3.37±0.06	2.96±0.09^NS^		2.99±0.09^NS^		3.02±0.17^NS^	NS
**Media thickness (µm)**							
SHR	202.4±3.8	178.4±5.1**		181.1±4.1**		178.6±1.7**	*<0.001*
WKY	131.5±7.6	131.7±5.6^NS^		131.6±2.0^NS^		131.1±5.3^NS^	NS

Values represent mean±SEM (n = 6).

One-way ANOVA was used to determine the significance levels on the effect of treatments compared with the corresponding control group (p_treatment_), NS = not significant.

NS , not significant; *p<0.05, **p<0.01, compared with strain-matched controls as assessed by Bonferroni’s Multiple Comparison Test.

SO = super olein; RPO = red palm olein; PO = palm olein.

### Effect of Palm Oil Treatments on the Heart and Aorta

Heart weights were reduced in SO (1.45±0.04 g), RPO (1.45±0.04 g) and PO (1.38±0.04 g) treated SHR compared with untreated SHR (1.60±0.11 g) but the reductions in heart weight were not significantly different from the SHR controls (Table 3). However, when the heart weight was expressed as a ratio to body weight, the reductions in heart weight were statistically significant in the SO (4.05±0.12) and PO (4.00±0.07) treated SHR (*p<0.05* in both cases) but not in the RPO treated SHR (4.44±0.12) compared with SHR controls (5.00±0.35) (Table 3).

In WKY rats, significant differences in heart weight were not observed between the SO (1.02±0.02 g), RPO (1.09±0.07 g) and PO (1.12±0.03 g) treated groups and the controls (1.12±0.02 g) (Table 3). Heart to body weight ratios were lower in SO (2.96±0.09), RPO (2.99±0.09) and PO (3.02±0.17) treated WKY rats but did not reach statistically significant levels compared with untreated WKY rats (3.37±0.06) (Table 3).

**Figure 3:**
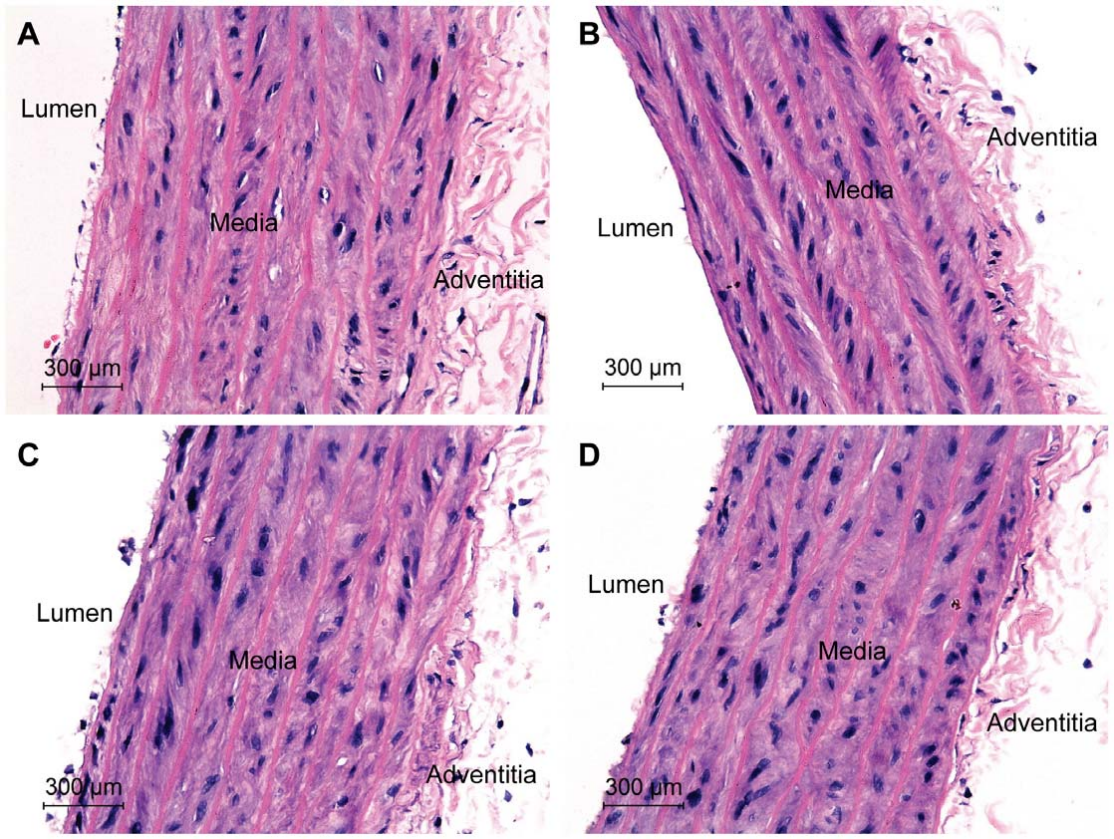
Photomicrograph of thoracic aorta from treated and untreated SHR rats at 400× magnification. Hematoxylin and eosin stained aorta: A. Control SHR. B. SO treated SHR. C. RPO treated SHR. D. PO treated SHR. Reduced aortic media thickness was observed in treated SHR compared with SHR controls.

**Figure 4:**
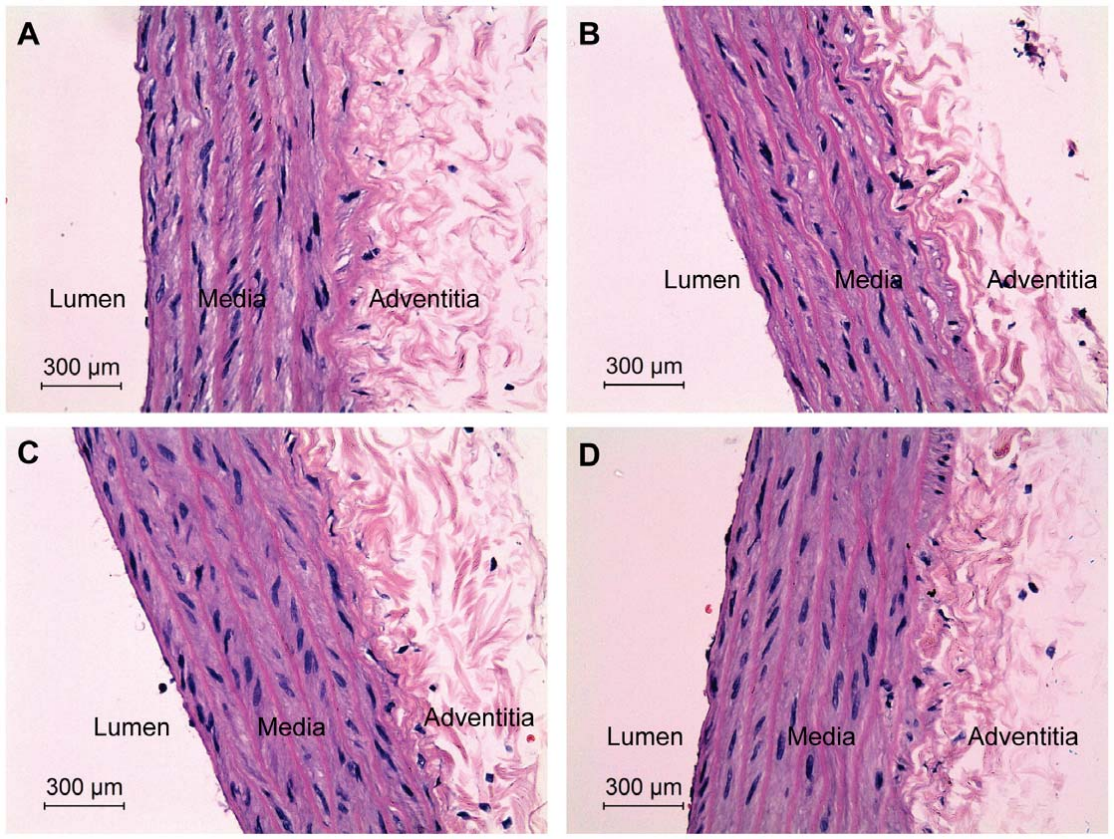
Photomicrograph of thoracic aorta from treated and untreated WKY rats at 400× magnification. Hematoxylin and eosin stained aorta: A. Control WKY. B. SO treated WKY. C. RPO treated WKY. D. PO treated WKY. No significant changes were observed in the aortic media thickness between the treated and control WKY rats.

Morphological differences in the aortic media thickness of treated and control SHR, and treated and control WKY rats are presented in Figures 3A–D and 4A–D, respectively. One-way ANOVA showed that treatments with SO, RPO and PO had a significant effect on the aortic media thickness (*p<0.001*) in the SHR and not in WKY rats (Table 3). The aortic media thickness was 202.4±3.8 µm in the SHR controls and was significantly reduced in SHR treated with SO (178.4±5.1 µm, *p<0.01*), RPO (181.1±4.1 µm, *p<0.01*) and PO (178.6±1.7 µm, *p<0.01*) (Table 3). On the other hand, no significant differences were observed between SO (131.7±5.6 µm), RPO (131.6±2.0 µm) and PO (131.1±5.3 µm) treated WKY rats compared with WKY controls (131.5±7.6 µm) (Table 3). Enface staining of the thoracic-abdominal aorta with Oil Red O did not show any lipid depositions in all the treated and untreated rats of both strains, and as such figures were not included here.

### Serum Lipid Profiles and Biochemical Indices of Liver and Renal Function

The levels of serum total cholesterol, triglycerides, HDL-C and LDL-C as well as total cholesterol/HDL ratio are presented in Table 4. Serum total cholesterol and triglyceride levels in SO, RPO and PO treated SHR were not significantly different from those of SHR controls. The HDL-C in SO (0.52±0.03 mmol/L), RPO (0.53±0.03 mmol/L) and PO (0.46±0.04 mmol/L) treated SHR were generally increased compared with untreated SHR (0.39±0.05 mmol/L) but did not reach statistical significance. The LDL-C was significantly reduced in both the SO (0.51±0.06 mmol/L) and RPO (0.51±0.08 mmol/L) treated SHR (*p<0.01* in both cases) but not in PO (0.71±0.03 mmol/L) treated SHR compared with untreated SHR (0.82±0.06 mmol/L). Accordingly, the total cholesterol/HDL ratio of SO (3.2±0.07), RPO (2.9±0.06) and PO (3.6±0.32) treated SHR were lower than those of untreated SHR (4.2±0.27), but were statistically significant for both the SO (*p<0.05*) and RPO (*p<0.01*) treated SHR and not for the PO treated group. On the other hand, serum lipid profile of SO, RPO and PO treated WKY did not differ significantly from the untreated WKY.

**Table 4:** Serum lipid profiles of treated and untreated SHR and WKY rats.

	Control	SO	RPO	PO
**Total Cholesterol (mmol/L)**				
SHR	1.6±0.14	1.7±0.02^NS^	1.6±0.08^NS^	1.6±0.04^NS^
WKY	2.6±0.13	2.5±0.28^NS^	2.2±0.23^NS^	2.3±0.16^NS^
**Triglycerides (mmol/L)**				
SHR	0.82±0.14	1.13±0.09^NS^	1.12±0.11^NS^	0.98±0.10^NS^
WKY	0.60±0.05	0.71±0.07^NS^	0.54±0.09^NS^	0.91±0.17^NS^
**HDL Cholesterol (mmol/L)**				
SHR	0.39±0.05	0.52±0.03^NS^	0.53±0.03^NS^	0.46±0.04^NS^
WKY	0.61±0.03	0.67±0.02^NS^	0.69±0.53^NS^	0.63±0.03^NS^
**LDL Cholesterol (mmol/L)**				
SHR	0.82±0.06	0.51±0.06**	0.51±0.08**	0.71±0.03^NS^
WKY	1.67±0.11	1.52±0.25^NS^	1.29±0.20^NS^	1.19±0.19^NS^
**Total Cholesterol/HDL ratio**				
SHR	4.2±0.27	3.2±0.07*	2.9±0.06**	3.6±0.32^NS^
WKY	4.2±0.05	3.7±0.32^NS^	3.3±0.26^NS^	3.6±0.25^NS^

Values represent mean±SEM (n = 6).

NS , not significant;

* *p<0.05*;

** *p<0.01* indicate a significant difference from strain-matched controls as assessed by Bonferroni's Multiple Comparison Test.

SO = super olein; RPO = red palm olein; PO = palm olein.

The levels of total protein, albumin, globulin, albumin/globulin ratio (AG ratio), total bilirubin, alkaline phosphatase (ALP), gamma glutamyl transferase (GGT), aspartate transaminase (AST) and alanine transaminase (ALT) in the serum of treated and untreated SHR and WKY rats representing the biochemical indices of liver function are presented in Table 5. Both SO, RPO and PO treated SHR and WKY rats showed no significant changes in the total protein, albumin and globulin levels as well as the AG ratio compared with strain-matched controls. ALP levels were increased in SO (363.7±33.5 U/L), RPO (427.0±44.3 U/L) and PO (283.5±21.0 U/L) treated SHR compared with untreated SHR (173.2±9.7 U/L). Significance differences were, however, observed in both the SO (*p<0.01*) and RPO (*p<0.001*) treated SHR and not in the PO treated SHR. However, the levels of AST and ALT in SO, RPO and PO treated SHR were not significantly different from those of untreated SHR. On the other hand, the levels of ALP (although generally increased), AST and ALT in SO, RPO and PO treated WKY were not significantly altered compared with WKY controls. The total bilirubin and GGT for all treated and untreated SHR and WKY rats were below detectable levels.

**Table 5:** Biochemical indices of liver function in treated and untreated SHR and WKY rats.

	Control	SO	RPO	PO
**Total protein (g/L)**				
SHR	63.7±0.8	61.7±0.2^NS^	62.3±1.0^NS^	62.3±1.2^NS^
WKY	60.8±1.1	63.0±1.8^NS^	66.3±1.7^NS^	66.0±1.1^NS^
**Albumin (g/L)**				
SHR	38.3±0.8	37.2±0.2^NS^	37.5±0.7^NS^	37.7±1.1^NS^
WKY	35.5±0.2	35.8±0.6^NS^	37.5±0.4^NS^	36.2±0.6^NS^
**Globulin (g/L)**				
SHR	25.3±0.4	24.5±0.2^NS^	24.8±0.4^NS^	24.7±0.5^NS^
WKY	25.3±1.0	27.2±1.4^NS^	29.7±1.3^NS^	29.8±0.9^NS^
**AG ratio**				
SHR	1.50±0.05	1.52±0.02^NS^	1.52±0.02^NS^	1.53±0.06^NS^
WKY	1.42±0.05	1.33±0.06^NS^	1.27±0.05^NS^	1.22±0.03^NS^
**ALP (U/L)**				
SHR	173.2±9.7	363.7±33.5**	427.0±44.3***	283.5±21.0^NS^
WKY	161.0±6.5	366.8±61.5^NS^	382.8±66.1^NS^	375.7±61.7^NS^
**Total bilirubin (µmol/L)**				
SHR	<2	<2	<2	<2
WKY	<2	<2	<2	<2
**GGT (U/L**)				
SHR	<3	<3	<3	<3
WKY	<3	<3	<3	<3
**AST (U/L)**				
SHR	155.0±14.2	153.0±6.5^NS^	175.5±9.3^NS^	142.5±11.4^NS^
WKY	127.5±10.6	131.5±10.7^NS^	138.5±7.3^NS^	144.8±8.5^NS^
**ALT (U/L)**				
SHR	63.5±4.7	71.2±3.4^NS^	76.7±3.2^NS^	60.83±2.5^NS^
WKY	59.8±2.7	64.2±3.0^NS^	58.5±2.7^NS^	59.8±3.3^NS^

Values represent mean±SEM (n = 6).

NS , not significant;

** *p<0.01*,

*** *p<0.001*, indicate a significant difference from strain-matched controls as assessed by Bonferroni's Multiple Comparison Test.

AG ratio = albumin/globulin ratio; ALP = alkaline phosphatase; GGT = gamma glutamyl transferase; AST = aspartate transaminase; ALT = alanine transaminase; SO = super olein; RPO = red palm olein; PO = palm olein.

Table 6 indicates the biochemical indices of renal function of SO, RPO and PO treated SHR and WKY rats as represented by the serum concentration of sodium, potassium, chloride, urea, creatinine and uric acid. The serum concentration of sodium, potassium, chloride, urea, creatinine and uric acid in SO, RPO and PO treated SHR and WKY rats were not significantly different compared with their corresponding controls.

**Table 6:** Biochemical indices of renal function in treated and untreated SHR and WKY rats.

	Control	SO	RPO	PO
**Sodium** **(mmol/L)**				
SHR	139.0±0.8	137.3±0.4^NS^	136.7±0.7^NS^	138.7±0.4^NS^
WKY	137.7±0.5	136.8±0.5^NS^	139.3±0.3^NS^	138.7±0.4^NS^
**Potassium** **(mmol/L)**				
SHR	5.6±0.3	5.3±0.1^NS^	5.7±0.3^NS^	5.9±0.2^NS^
WKY	5.8±0.2	5.3±0.2^NS^	5.2±0.2^NS^	5.4±0.1^NS^
**Chloride** **(mmol/L)**				
SHR	99.7±1.0	97.7±1.0^NS^	97.7±0.8^NS^	101.2±0.3^NS^
WKY	100.7±0.2	101.5±0.8^NS^	101.2±0.7^NS^	102.0±0.5^NS^
**Urea** **(mmol/L)**				
SHR	8.4±0.5	8.7±0.3^NS^	9.7±0.5^NS^	8.3±0.5^NS^
WKY	7.0±0.4	7.0±0.5^NS^	7.1±0.4^NS^	6.7±0.3^NS^
**Creatinine** **(µmol/L)**				
SHR	32.5±1.3	28.7±0.9^NS^	33.7±1.9^NS^	33.2±2.4^NS^
WKY	29.0±1.3	33.2±1.7^NS^	34.2±1.7^NS^	34.0±2.0^NS^
**Uric acid** **(mmol/L)**				
SHR	0.067±0.006	0.072±0.008^NS^	0.078±0.010^NS^	0.083±0.008^NS^
WKY	0.055±0.003	0.067±0.003^NS^	0.072±0.007^NS^	0.060±0.005^NS^

Values represent mean±SEM (n = 6).

NS , not significant, compared with strain-matched controls as assessed by Bonferroni's Multiple Comparison Test.

SO = super olein; RPO = red palm olein; PO = palm olein.

### Fatty Acid Composition and Phytonutrient Content of SO, RPO and PO

The SO, RPO and PO fatty acid composition and phytonutrient content are shown in Table 1. Overall, SO, RPO and PO possess the same major fatty acids but differ slightly in their fatty acid composition and phytonutrient content. The SO exhibits the highest oleic acid content of 45.4% followed by RPO (43.4%) and PO (42.2%). The palmitic acid content is the lowest in SO (35.6%), and is higher with approximately equal amount in both PO (40.3%) and RPO (39.5%). Similarly, the unsaturated to saturated fatty acid ratio is the highest in the SO (1.47) compared with RPO (1.25) and PO (1.18). The RPO contains the highest carotene and vitamin E content at 564.0 mg/kg and 1141.0 mg/kg, respectively, while SO has the least (7.0 mg/kg carotene and 188.0 mg/kg vitamin E).

## Discussion

The most common form of human hypertension is the primary or essential hypertension and is best represented by the genetic animal models of hypertension such as the SHR [16]. SHR models essential hypertension in many respects and follows the similar progression of hypertension from pre-hypertensive phase to developing and sustained hypertensive phases as in human essential hypertension [12], [17]. In the SHR, BP rises progressively from four weeks of age, with each phase lasting for at least more than a few weeks. Therefore in this study, SHR of four weeks of age was used as it enables us to observe the time course of effect of long-term intake of oil-supplemented diets on the progression of hypertension. Other genetic hypertensive strains of rats, of which many reflect a subgroup of essential hypertensive, such as the Dahl salt-sensitive rats [16] and Milan hypertensive rats [18] which develop hypertension based on salt-sensitivity or sodium retention, are not appropriate models for our study. In this study, male SHR rats instead of the females were used to eliminate the influence of sex hormones on the BP [19]. We have also conducted similar experiments with male WKY rats which served as the normotensive control strain for SHR [16] to evaluate the possible effects of SO, RPO and PO in these animals.

Normally, palm oil has approximately equal fraction of saturated fatty acid to that of unsaturated fatty acid [2], but the SO, RPO and PO used in this study have increased unsaturated fatty acid to saturated fatty acid content. Total unsaturated fatty acid made up 59.6% in SO, 55.6% in RPO and 54.1% in PO, owing to the advances in palm oil processing technology. SO, RPO and PO are naturally fortified with antioxidants, mainly vitamin E and carotenes (Table 1).

In our study, SHR and WKY rats were fed with diet containing 15% wt/wt SO, RPO or PO where 34.7% of the total energy content was provided by fat, and this was similar and comparable to most human diets which contain 30–40% of the total energy content provided by fat [20]. As the fat content provided in the diets for the treated groups was higher than that of the controls, food intake was measured as calorie intake. We found that the calorie intake of SO, RPO and PO treated SHR and WKY rats were similar to that of their strain-matched controls (Figs. 2A and 2B). Because of these findings, body weight was used to represent the weekly growth for all rats. Since our data showed that the body weight gain was similar amongst the treated and control groups (Figs. 2C and 2D), it therefore appears that dietary supplementation with 15% SO, RPO and PO had no influence on the normal growth pattern of these animals.

The amount of sodium present in commercial rodent diets may raise concern. High sodium diet raises BP though such rise in BP augments renal sodium excretion and regains normal sodium balance. However, sodium retention coupled with high sodium intake can trigger a rise in BP in both susceptible humans and animals [21]. The standard diet for control animals which was used to formulate the 15% palm oil supplemented diets sourced from Specialty Feeds, Australia contains only 0.18% sodium wt/wt. Sodium content of 0.18% wt/wt is well below most commercial rodent diets which may contain 0.3–0.5% sodium which is about sevenfold higher than that required for normal growth of rats [22].

The amount of sodium present in the standard and formulated rat diets used in this study may still pose some concerns as to whether this may affect the outcome of our results, since the SO, RPO and PO diets formulated based on 15% wt/wt oil with standard rat diet contain a lower sodium content. The findings of Aoki et al. [23] clearly underscored the lack of influence of dietary sodium on the development and degree of hypertension in SHR on low (0.079%), standard (0.276%) and high (2.76%) sodium diets from 5 weeks up to 40 weeks of age. These findings were confirmed in subsequent studies in SHR on 0.05% and 0.29% sodium diets from 7 to 10 weeks of age [24] and 0.06%, 0.10% and 0.23% sodium diets from birth up to 16 weeks of age with all diet groups exhibiting similar growth rates [25] as well as 0.05% and 0.4% sodium diets from 4 to 30 weeks of age with no effect on normal growth [26]. In contrast, however, 3.4% high sodium diet and 3% high salt diet (1.2% Na wt/wt) have been found to significantly exacerbate the blood pressure of SHR [24], [27]. On the other hand, the rise in blood pressure of SHR can be prevented when the dietary sodium level falls below 0.05% w/wt. This has been demonstrated in SHR fed with 0.02% and 0.04% sodium diets from birth up to 16 weeks of age [25] and 0.1% salt (0.04% Na wt/wt) diet from 4 weeks until 6 months of age [27]. However, these dietary sodium levels are so low that it affects the overall growth [25], [27]. The minimum dietary sodium requirement of the rat for normal growth has been shown to be 0.05% wt/wt [28]. In our study, the sodium content of the standard (0.18% wt/wt) and formulated (0.15% wt/wt) diets were invariably within the range of dietary sodium reported by others to have no effect on the development and degree of hypertension as well as the normal growth in SHR. Hence, it is unlikely that the reduction, albeit minute, in the average daily sodium intake in SO, RPO and PO treated SHR (0.0245±0.0007 g, 0.0252±0.0007 g and 0.0262±0.0004 g, respectively) compared with SHR controls (0.0333±0.0010 g) is the cause of the prevention in the rise of blood pressure in treated SHR observed in this study.

### Effect of Palm Oil Treatments on Systolic Blood Pressure and Heart Rate

In this study, we demonstrated that the rapid and spontaneous rise in the SBP that occurs in the untreated SHR with age was effectively mitigated by dietary supplementation with SO, RPO and PO (Fig. 1A). These palm oils induced marked reductions in the SBP of SHR by the end of 15 weeks of treatment (Table 2). These findings suggest that the antihypertensive effect of palm oil is not confined to a specific type of the oil as all of the oils tested were able to delay the onset and suppress the progression of hypertension.The SHR demonstrated a greater sensitivity to the depressor effects of SO and PO than did RPO (Table 2). However, we are not able at this stage to discriminate the reason by which SO, RPO and PO prevent the rise in BP, although it is possible that the oleic acid and vitamin E present in these oils may be responsible for the relative degree in the hypotensive responses induced by these oils. It has been reported that the fatty acid composition of membrane phospholipids can be profoundly altered by changes in the dietary lipid intake [29]. The ratio of polyunsaturated fatty acid or monounsaturated fatty acid to saturated fatty acid in membrane lipids influences the membrane fluidity, and membranes tend to become more fluid with higher unsaturated to saturated fatty acid ratio [30]. Perona et al. [30] reported that high amount of oleic acid present in the olive oil may be responsible for the proportional increase of oleic acid in membrane phospholipids in olive oil treated SHR. Changes in the membrane lipid structure may affect the contractility of smooth muscles to influence the BP and HR [29]. These studies raised the possibility that substantial amount of unsaturated fatty acids, in particular the oleic acid present in SO (45.4%), RPO (43.4%) and PO (42.2%) (Table 1), may produce similar changes to the membrane lipid structure that prevented the rapid rise in the BP of SHR treated groups.

Oleic acid has been shown to lower the BP [9], [31] by enhancing the vasodilatory α_2A/D_-adrenoreceptor/G protein/adenylyl cyclase-cAMP/PKA pathway [32], while limiting the vasoconstriction pathways such as the inositol-triphosphate, Ca^2+^, diacylglycerol and Rho kinase [9]. It is possible that the observed antihypertensive effects of SO, RPO and PO in SHR are mediated through the actions of oleic acid in the blood vessels on these pathways. Docosahexaenoic acid, linolenic acid and linoleic acid have been shown to directly stimulate two-pore domain potassium channel (TREK/TRAAK channels) which causes hyperpolarization and promotes vascular relaxation [33]. Palm oil, being rich in polyunsaturated fatty acids especially linoleic acid (13.6% in SO, 12.2% in RPO and 11.3% in PO, Table 1), may partly inhibit the rise of BP in the SHR by modulating the TREK/TRAAK channels. Palm oil has also been reported to elevate the aortic cGMP that mediates the release of nitric oxide from endothelial cells which, in turn, stimulates the guanylate cyclase in the vascular smooth muscle cells and causes vascular relaxations [29].

Palm oil also possesses abundant health promoting phytonutrients especially vitamin E and carotenes [34]. Antioxidant status has been found to be closely associated with the cardiovascular pathologies [35], [36]. Vitamin E may prevent the development of hypertension, reduce lipid peroxides in the plasma and blood vessels, and enhance total antioxidant status [37], [38] while higher circulating level of carotenoids was associated with a lower risk of hypertension [39]. Thus, the antioxidants present in SO, RPO and PO (Table 1) may contribute, at least partly, to the BP regulation.

Bradycardia was associated with the fall in BP towards the end of the treatment period in SO and PO treated SHR (Table 2). It is interesting to note that RPO has little effect on the HR in the SHR. The bradycardia observed in the SO and PO treated SHR may be attributable to a reduction in the sympathetic tone [40]. The SHR strain is a hypertensive rat model with increased sympathetic tone [41] and the increased sympathetic activity in the SHR is associated with increased HR in these animals [42]. There is increasing evidence that many forms of human hypertension are initiated and maintained by an elevated sympathetic tone [43], [44].Both the SBP (Fig. 1C and Table 2) and HR (Fig. 1D and Table 2) of WKY rats were not affected by SO, RPO and PO treatments, suggesting that the homeostatic mechanisms regulating the BP and HR in these normotensive animals were not altered following treatments with these oils.

### Effect of Palm Oil Treatments on the Heart and Aorta

Cardiac hypertrophy is one of the features of hypertensive end-organ damage observed in the SHR and human essential hypertensives [45], and can be reverted by hypotensive treatment [42]. The increase in cardiac mass has been shown to be driven not only by increased cardiac load associated with increased BP but also by increased cardiac sympathetic tone [46], [47]. In view of these observations, we therefore evaluated the heart weight and heart to body weight ratio of treated and untreated animals.

Our data showed that after 15 weeks of experiment, the heart weight and heart to body weight ratio of the SHR controls were higher than the normotensive WKY controls (Table 3). This is in agreement with the findings by Jespersen et al. [42] that a large increase in total heart weight in the SHR occurs with age concurrently with the rise in BP from four-week-old to maturity. We also showed that the heart to body weight ratio of SHR was reduced following treatments with SO and PO. These data indicate that the SO and PO have the potential to prevent the development of cardiac hypertrophy by repressing the spontaneous rise of BP in the SHR, possibly as a result of reduced sympathetic activity and cardiac load [46], [47]. Interestingly, the heart weight and heart to body weight ratio in the WKY rats were not affected by treatments with SO, RPO and PO (Table 3).

Hypertrophy and tissue disorganization are not limited to the myocardium but also involve vascular smooth muscle cells [48]. In hypertension, the basic haemodynamic abnormality is increased vascular resistance, which is due not only to vasoconstriction, but also to structural changes in the arterial wall [49]. The increase in media thickness is a marker of structural modifications characterized by smooth muscle cell hypertrophy and fibrosis which is directly related to the higher pressure and wall stress levels [50], [51]. Disorganization of the media may include changes in the number of muscle to elastic lamellae connections, an increase in the ratio of collagen type I to III or an accumulation of advanced glycation end products. Mechanical alteration of the aortic wall occurs earlier in hypertensive than in normotensive rats [51].

In our study, SHR controls exhibited significant aortic media thickening with evidence of increased muscle to elastic lamellae connections (Fig. 3 and Table 3). SO, RPO and PO treated SHR showed reduced aortic media thickening with reduced interlamellar space (Fig. 3 and Table 3) which may be consequential to the reduction in BP. The structural changes in the arterial wall observed between treated and untreated SHR was restricted to aortic media thickening marked by the space between the lamellas as the number of the lamellas was unchanged. There were no significant pathologies observed in the aortas of both treated and untreated SHR. In addition, no significant structural changes and pathologies in the arterial wall were observed between the SO, RPO and PO treated WKY rats and WKY controls (Fig. 4).

### Serum Lipid Profiles and Biochemical Indices of Liver and Renal Function

Despite the fractionation process in palm oil processing which brought about an increase in monounsaturated oleic acid with concurrent reduction in palmitic acid [5], the palmitic acid is still present in considerable amount in SO (35.0%), RPO (39.5%) and PO (40.3%) (Table 1). Palmitic acid is regarded as the major cholesterol raising saturated fatty acid in the diet [52]. Therefore, it was generally assumed that elevations of total cholesterol and LDL-C are predictable following long-term consumption of palm oil. These parameters are positively correlated with the incidence of cardiovascular disease [6].

We showed that SO, RPO and PO treated SHR and WKY rats produced similar trend in their serum lipid profiles. Our data argue against the notion that long-term consumption of palm oil elevates the total cholesterol and LDL-C levels since the treated animals did not show any changes in the total cholesterol, and the LDL-C level was actually reduced with SO and RPO in the SHR (Table 4). These findings are consistent with that reported by Kris-Etherton et al. [53] in rats fed with 10% wt/wt palm oil diet. Other studies have also reported reduced LDL-C levels with palm oil diets [1], [54]. Overall, we showed that long-term dietary intake of SO, RPO and PO have the propensity to raise the HDL-C levels in the SHR. These findings are consistent with the previously reported studies [54], [55]. Meanwhile, total cholesterol/HDL ratio was decreased in the SO and RPO treated SHR (Table 4) suggesting that these oils reduced the atherogenic index of the animals. These data were consistent with the previous reports that palm oil reduces the risk of atherogenesis [1], [56]. As rats are known to be exceptionally resistant to atherosclerosis unless fed with extremely atherogenic diet [45], this explained the lack of lipid depositions with the enface staining of thoracic-abdominal aorta with Oil Red O in our study.

We found that the biochemical indices of liver function were not affected following treatments with SO, RPO and PO in both the SHR and WKY rats, with the exception that the ALP levels were increased in the SHR treated groups. There was a tendency for the ALP levels to increase in the WKY treated rats. The elevated serum ALP levels may indicate cholestatic disorders or obstructive liver disease such as intrahepatic and extrahepatic obstruction to bile flow as well as extrahepatic biliary atresia although its precise roles are unknown [57]. On the other hand, elevated serum ALP levels was also reported by Owu et al. [58] in WKY rats fed with fresh palm oil, and they concluded that the low levels of oxidation in fresh palm oil due to the presence of high amounts of antioxidant caused little injury to the liver. Fat ingestion has been shown to elevate serum ALP levels in both healthy humans and animals either acutely or chronically [59]. This indicates that the elevation of serum ALP levels following the consumption of SO, RPO and PO in the SHR in our experiments is an expected phenomenon. In addition, the liver histological sections obtained from both of these rat strains (photomicrographs not shown) did not show any gross morphological changes at the cellular level between treated and untreated groups. This evidence further argues against any contention for liver injury or liver diseases as indicated by the elevated levels of serum ALP.

After 15 weeks of experiment, the biochemical indices of renal function were determined for urea, creatinine and electrolytes to assess the functional capacities of the kidneys. Our results showed that long-term treatments with SO, RPO and PO did not produce any significant variations in the renal biochemical indices in both the SHR and WKY rats (Table 6). In spite of the probability that renal abnormalities are present in animals susceptible to hypertension [21], [60], such abnormalities were unlikely to be evident in the 19-week-old rats at the end of 15 weeks of experiment in our study, as the urinary and fractional excretion of sodium in the SHR have been shown not to differ from that of WKY rats for up to 52 weeks of age [61].

### Conclusion

In conclusion, our findings suggest that these oils can provide effective dietary interventions aimed at improved control of blood pressure in the treatment of hypertension. These oils also reduce the risk of cardiovascular disease associated with hypertension by improving the serum lipoprotein profiles, and cardiac and vascular smooth muscle hypertrophy produced by hypertension.
